# Clustered Genes Encoding 2-Keto-l-Gulonate Reductase and l-Idonate 5-Dehydrogenase in the Novel Fungal d-Glucuronic Acid Pathway

**DOI:** 10.3389/fmicb.2017.00225

**Published:** 2017-02-14

**Authors:** Joosu Kuivanen, Mikko Arvas, Peter Richard

**Affiliations:** VTT Technical Research Centre of Finland LtdEspoo, Finland

**Keywords:** fungi, *Aspergillus*, metabolism, D-glucuronate, D-glucuronic acid, L-idonate, 2-keto-L-gulonate

## Abstract

D-Glucuronic acid is a biomass component that occurs in plant cell wall polysaccharides and is catabolized by saprotrophic microorganisms including fungi. A pathway for D-glucuronic acid catabolism in fungal microorganisms is only partly known. In the filamentous fungus *Aspergillus niger*, the enzymes that are known to be part of the pathway are the NADPH requiring D-glucuronic acid reductase forming L-gulonate and the NADH requiring 2-keto-L-gulonate reductase that forms L-idonate. With the aid of RNA sequencing we identified two more enzymes of the pathway. The first is a NADPH requiring 2-keto-L-gulonate reductase that forms L-idonate, GluD. The second is a NAD^+^ requiring L-idonate 5-dehydrogenase forming 5-keto-gluconate, GluE. The genes coding for these two enzymes are clustered and share the same bidirectional promoter. The GluD is an enzyme with a strict requirement for NADP^+^/NADPH as cofactors. The k_cat_ for 2-keto-L-gulonate and L-idonate is 21.4 and 1.1 s^-1^, and the K_m_ 25.3 and 12.6 mM, respectively, when using the purified protein. In contrast, the GluE has a strict requirement for NAD^+^/NADH. The k_cat_ for L-idonate and 5-keto-D-gluconate is 5.5 and 7.2 s^-1^, and the K_m_ 30.9 and 8.4 mM, respectively. These values also refer to the purified protein. The *gluD* deletion resulted in accumulation of 2-keto-L-gulonate in the liquid cultivation while the *gluE* deletion resulted in reduced growth and cessation of the D-glucuronic acid catabolism.

## Introduction

The genus *Aspergillus* is a large group of filamentous fungi containing species that are known to be versatile decomposers of biomass polymers (de Vries and Visser, 2001). *Aspergillus niger* – a member of the group of black aspergilli – is widely used in industrial biotechnology due to its useful characteristics such as capacity to produce organic acids and biomass hydrolysing enzymes in high yields. Several different sugars and sugar acids resulting from the extracellular biomass hydrolysis by a mixture of secreted enzymes are catabolized by the organism through metabolic pathways. Many of these pathways are known and characterized; however, some remain still unknown and may contain enzymes and biochemical reactions that are not described earlier. These reactions may serve as source of enzymes for biotechnological applications such as production of fuels and chemicals from biomass.

**FIGURE 1:**
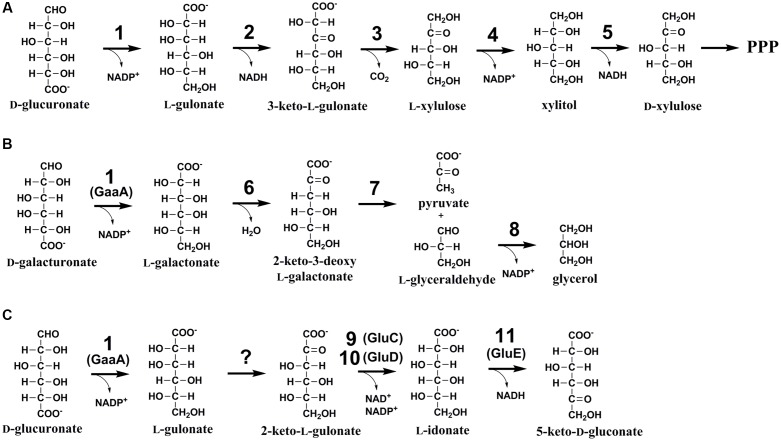
**(A)** The catabolic D-glucuronic acid pathway in animals, **(B)** the fungal D-galacturonic acid pathway, and **(C)** the first suggested reactions in the fungal D-glucuronic acid pathway. The enzymes are: (1) hexuronate reductase, (2) L-gulonate 3-dehydrogenase, (3) 3-keto-L-gulonate decarboxylase, (4) L-xylulose reductase, (5) xylitol dehydrogenase, (6) L-galactonate dehydratase, (7) 2-keto-3-deoxy-L-galactonate aldolase, (8) L-glyceraldehyde reductase, (9) NADH dependent 2-keto-L-gulonate reductase, GluC, (10) NADPH dependent 2-keto-L-gulonate reductase, GluD (in this study), and (11) L-idonate dehydrogenase, GluE (in this study).

One such a biomass component with limited knowledge on its catabolism is D-glucuronic acid (D-glcUA). It occurs in the cell wall polysaccharides such as glucuronoxylan (Reis et al., 1994) in plants and ulvan (Lahaye and Robic, 2007) in algae. In nature, D-glcUA resulting from biomass hydrolysis is catabolised by saprotrophic microorganisms through different metabolic pathways. In bacteria, two different catabolic pathways for D-glcUA are known: an isomerase pathway (Ashwell, 1962) and an oxidative pathway (Dagley and Trudgill, 1965; Chang and Feingold, 1970). D-GlcUA and its close structural isomer D-galacturonic acid (D-galUA), a pectin constituent, are catabolized analogously via these pathways in bacteria. Some of the enzymes in these pathways have dual functions and are used for the catabolism of both compounds. In addition to the bacterial pathways, a different catabolic D-glcUA pathway is known in animal cells (Hankes et al., 1969). The animal pathway, also known as glucuronate-xylulose-pentose phosphate pathway or uronate cycle, contains two reduction, two oxidation and one decarboxylation reactions resulting in formation of D-xylulose, which, after phosphorylation to D-xylulose 5-phosphate, is a metabolite of pentose phosphate pathway (**Figure 1A**). In fungi, the catabolic pathway for D-galUA is well known including reduction, dehydration, an aldolase reaction and second reduction (**Figure 1B**) (Kuorelahti et al., 2005, 2006; Liepins et al., 2006; Hilditch et al., 2007). However, a fungal pathway for D-glcUA catabolism is only partly known.

The first enzyme for D-galUA catabolism in the filamentous fungus *A. niger* has most likely a dual function and is also the first enzyme in D-glcUA catabolism. The *gaaA*, encoding a hexuronate reductase is reducing D-galUA to L-galactonate and D-glcUA to L-gluconate (Martens-Uzunova and Schaap, 2008; Kuivanen et al., 2016). Transcription of *gaaA* was induced on both of these carbon sources and deletion of the gene reduced the catabolism of both carbon sources, however, did not block it completely (Kuivanen et al., 2016). In the following steps the pathways for D-galUA and D-glcUA differ, the L-galactonate dehydratase showed no activity with L-gulonate and an L-gulonate dehydratase activity was not found in *A. niger* (Motter et al., 2014). An enzyme that is essential for D-glcUA catabolism was identified to be a NADH dependent 2-keto-L-gulonate reductase, GluC (Kuivanen et al., 2016). Deletion of *gluC* gene resulted in reduced growth on D-glcUA plates and blocked the D-glcUA consumption in liquid cultivations. The L-gulonate is converted to 2-keto-L-gulonate by an unknown activity. For the further conversion of L-idonate, two enzyme activities have been described in the literature: the NAD^+^ and NADP^+^ dependent L-idonate 5-dehydrogenases (EC 1.1.1.366 and EC 1.1.1.264). The NAD^+^ dependent activity has been described in plants (Wen et al., 2010) in the pathway for L-ascorbic acid catabolism (DeBolt et al., 2006) and in bacteria as part of L-idonate catabolism (Bausch et al., 1998). The NADP^+^ dependent L-idonate 5-dehydrogenase activity was described for the first time already long time ago in the filamentous fungus *Fusarium* sp. (Takagi, 1962). However, there is no report on a fungal L-idonate 5-dehydrogenase gene or the biological function of such a gene.

In the present study, we identify a gene cluster encoding NADPH dependent, L-idonate forming, 2-keto-L-gulonate reductase and NAD^+^ dependent L-idonate 5-dehydrogenase which forms 5-keto-D-gluconate (**Figure 1C**). These genes are involved in the fungal D-glcUA catabolism and the reaction catalyzed by the latter enzyme is a direct continuation for the previously identified reaction by the action of GluC.

## Materials and Methods

### Strains

The *A. niger* strain ATCC 1015 (CBS 113.46) was used as a wild type. The *A. niger* mutant strain Δ*pyrG* (deleted orotidine-5′-phosphate decarboxylase) was described earlier (Mojzita et al., 2010). All the plasmids were produced in *Escherichia coli* TOP10 cells. The *Saccharomyces cerevisiae* strains ATCC 90845 and a modified CEN.PK2 (*MATα, leu2-3/112, ura3-52, trp1-289, his3-*Δ*1, MAL2-8^c^, SUC2*) were used in the homologous recombination for the plasmid construction and for the production of the purified GluD and GluE enzymes, respectively.

### Media and Cultural Conditions

Luria Broth culture medium supplemented with 100 μg ml^-1^ of ampicillin and cultural conditions of 37°C and 250 rpm were used with *E. coli*. YPD medium (10 g yeast extract l^-1^, 20 g peptone l^-1^, and 20 g D-glucose l^-1^) was used for yeast pre-cultures. After the transformation of an expression plasmid in yeast, SCD-URA (uracil deficient synthetic complete media supplemented with 20 g D-glucose l^-1^) plates were used for uracil auxotrophic selection. SCD-URA medium was used in protein production. All the yeast cultivations were carried out at 30°C and the liquid cultivations at 250 rpm. *A. niger* spores were generated on potato-dextrose plates and ∼10^8^ spores were inoculated to 50 ml of YP medium (10 g yeast extract l^-1^, 20 g peptone l^-1^) containing 30 g gelatin l^-1^ for pre-cultures. Mycelia were pre-grown in 250-ml Erlenmeyer flasks by incubating overnight at 28°C, 200 rpm and harvested by vacuum filtration, rinsed with sterile water and weighted. In *A. niger* transformations, SCD-URA plates supplemented with 1.2 M D-sorbitol and 20 g agar l^-1^ (pH 6.5) were used. *A. nidulans* defined minimal medium (Barratt et al., 1965) was used in the *A. niger* cultivations. The minimal medium used in the phenotypic characterization in liquid cultivations contained 20 g D-glcUA l^-1^ and the pH was adjusted to 3. These cultivations were inoculated with 20 g l^-1^ (wet) of pre-grown mycelia. The strain Δ*gluD* was cultivated in 24-well plates in 4 ml final volume and Δ*gluE* in shake flasks in 25 ml final volume. Agar plates used for phenotypic characterization contained uracil deficient SC-medium (synthetic complete), 15 g agar l^-1^ and 10 g D-glcUA l^-1^. Plates were inoculated with 1.5^∗^10^6^ spores and incubated at 28°C for 3 days.

### Transcriptional Analysis

The RNA sequencing for transcriptional analysis was carried out as described previously (Kuivanen et al., 2016). The protein ID numbers refer the numbers from the Join Genome Institute (JGI), MycoCosm, *A. niger* ATCC 1015 v.4.0 database, available at: http://genome.jgi.doe.gov/Aspni7/Aspni7.home.html.

### Protein Production and Purification

The gene *gluD* was amplified by PCR (KAPA HiFi DNA polymerase, Kapa Biosystems, primers in **Table 1**) from *A. niger* cDNA extracted and generated from D-glcUA cultivated wild type strain. The resulting DNA fragment was digested with *BamHI* and *NheI* (both NEB) and ligated into a modified pYX212 plasmid (Verho et al., 2004) containing *TPI1* promoter and *URA3* selectable marker. The *gluE* gene was custom synthesized as a yeast codon optimized gene (GenScript, USA), released with *EcoRI* and *BamHI* (both NEB) and ligated into the modified pYX212 plasmid. For the histidine-tagged protein, *gluE* was amplified by PCR (primers in **Table 1**) and ligated in a similar manner to the modified pYX212 plasmid. A yeast strain was then transformed with the resulting plasmids using the lithium acetate method (Gietz and Schiestl, 2007). The procedure for protein production and purification was described previously (Kuivanen et al., 2016).

**Table 1:** Oligonucleotides used in the study.

Name	Sequence	Description
P1	ATATACATATGCGGGGTTCTGATGAATGTATGGGAGGTG	5′ flank of *gluD*, FOR
P2	TCAATCACCCGATGGCGATGATGTCAAG	5′ flank of *gluD*, REV
P3	GCGTGATAACATGTACTGTGACAGATACCAAC	3′ flank of *gluD*, FOR
P4	CCGGATCAAGCTTCGAATTCAGAGTTCAGGTCTGTTCTGTTC	3′ flank of *gluD*, REV
P5	CCCCCCCTCGAGGTCGACGGTATCGATAAGCTTGATATCGGCGGCCGCAGCCCAGCAG ACCATACCTA	5′ flank of *gluE*, FOR
P6	CTGGTATAGCCAAACATCGCCAATCACCTCAATCACCCGGGATGAATGTATGGGAGGTGT	5′ flank of *gluE*, REV
P7	GCCATGCGGGCTTGGGACGCCATGTCCGTCGCGTGATAACGTCGATGGCTGAATCTAATT	3′ flank of *gluE*, FOR
P8	AGCTCCACCGCGGTGGCGGCCGCTCTAGAACTAGTGGATCGCGGCCGCCTTTGGTAC TTCCGAGCCAG	3′ flank of *gluE*, REV
P9	GATGAATGTATGGGAGGTGT	amplification of *gluD* cassette, FOR
P10	AGAGTTCAGGTCTGTTCTGT	amplification of *gluD* cassette, REV
P11	CTAGGCGGAGATCTTTGGCG	colony PCR *gluD*, integration of the donor DNA, FOR
P12	AGCTGGTATAGCCAAACATC	colony PCR *gluD*, integration of the donor DNA, REV
P13	GTTGTAGGAGGTGGCTCGTC	colony PCR *gluD*, ORF of *gluD*, FOR
P14	GCACCAAAGTAATCATCTTC	colony PCR *gluD*, ORF of *gluD*, REV
P15	CAATGGCGGGTTCATTCTCA	colony PCR *gluE*, integration of the donor DNA, FOR
P16	AGCTGGTATAGCCAAACATC	colony PCR *gluE*, integration of the donor DNA, REV
P17	ACACCTCCCATACATTCATC	colony PCR *gluE*, ORF of *gluE*, FOR
P18	AATTAGATTCAGCCATCGAC	colony PCR *gluE*, ORF of *gluE*, REV
P19	TATAGGATCCACCATGCCCGCCGCATTGCTGAT	amplification of *gluD* from cDNA, FOR
P20	TATAGCTAGCCTATTTTCCAAACACATCCTTC	amplification of *gluD* from cDNA, REV
P21	TATAGGATCCACCATGCATCACCATCACCATCACGGTGGCGGTATGCCCGCCGCATTGCTGAT	amplification of *gluD* with His-tag, FOR
P22	TATAGAATTCACCATGCATCACCATCACCATCACGGTGGCGGTATGACAGTAAACTCCACAGA	amplification of *gluE* with His-tag, FOR
P23	TATAGGATCCTCAATCGTCACCACTTTCTA	amplification of *gluE* with His-tag, REV

### Enzymatic Assays

The oxidoreductase activity of purified GluD and GluE proteins was assayed using Konelab 20XT Clinical Chemistry Analyzer (Thermo Scientific). The reaction mixture contained 50 mM Tris buffer, 400 μM NAD+ or NADH, a substrate in different concentrations and purified proteins in a final concentration of 3.6 mg l^-1^. The pH 8 was used with NAD+ and L-idonate and pH 7 with NADH and 2-keto-L-gulonate and 5-keto-D-gluconate. The reaction was started by addition of the purified protein and the formation/consumption of NADH was followed at 340 nm. The kinetic parameters were determined using the IC50 tool kit^1^. L-Idonate and 2-keto-L-gulonate were ordered as custom synthesized by Omicron Biochemicals Inc, USA while 5-keto-D-gluconate was ordered from Sigma-Aldrich.

### Gene Deletions in *A. niger*

The deletion cassette for *gluD* contained homologous 5′ (∼450 bp) and 3′ flanks (∼650 bp) for targeted integration and the selectable marker *pyrG*. The 5′ and 3′ flanks were amplified by PCR with the primers as described in **Table 1**. The resulting PCR amplified fragments contained 40 bp compatible ends for homologous recombination with the *A. niger pyrG* and *EcoRI* and *BamHI* digested pRS426. The deletion cassette for *gluE* was constructed in a similar manner but contained homologous 5′ and 3′ flanks of 1.5 kb (primers in **Table 1**). All the fragments were joined using yeast homologous recombination as described earlier (Kuivanen et al., 2015). The resulting deletion cassette for *gluD* was produced by PCR amplification (primers in **Table 1**) from the resulting plasmid and the cassette for *gluE* deletion was produced by linearization of the plasmid with *NotI* (NEB). The *gluD* deletion cassette was transformed to *A. niger*Δ*pyrG* strain together with the CRISPR plasmid pFC-332 (Nodvig et al., 2015) and the *in vitro* synthesized sgRNA (CTCCTCCATCCTGACCTTGA) (GeneArt^TM^ Precision Synthesis Kit). The *gluE* deletion cassette was transformed to *A. niger*Δ*pyrG* strain without the CRISPR plasmid. Mutants with successful integration of the cassette were selected for growth in the absence of uracil and, in the case of *gluD* deletion, in the presence of hygromycin (for pFC-332) and in the absence of uracil (for the deletion cassette containing *pyrG*). Resulting transformants were screened for the correct integration of the deletion cassette and for the deletion of *gluD* or *gluE* open reading frame using diagnostic PCR (Phire direct PCR kit, Thermo Scientific, primers in **Table 1**).

### Chemical Analyses

Samples were removed from liquid cultivations at intervals and mycelium was separated from the supernatant by centrifugation or filtration. The concentration of D-glcUA and 2-keto-L-gulonate was determined by HPLC using a Fast Acid Analysis Column (100 mm × 7.8 mm, Bio-Rad Laboratories, Hercules, CA, USA) linked to an Aminex HPX-87H organic acid analysis column (300 mm × 7.8 mm, Bio-Rad Laboratories) with 5.0 mM H_2_SO_4_ as eluent and a flow rate of 0.5 ml min^-1^. The column was maintained at 55°C. Peaks were detected using a Waters 2487 dual wavelength UV (210 nm) detector. The retention times of the peaks resulting from the supernatant were compared with the retention times of standards.

## Results

### Clustered Genes Are Induced by D-Glucuronic Acid

**FIGURE 2:**
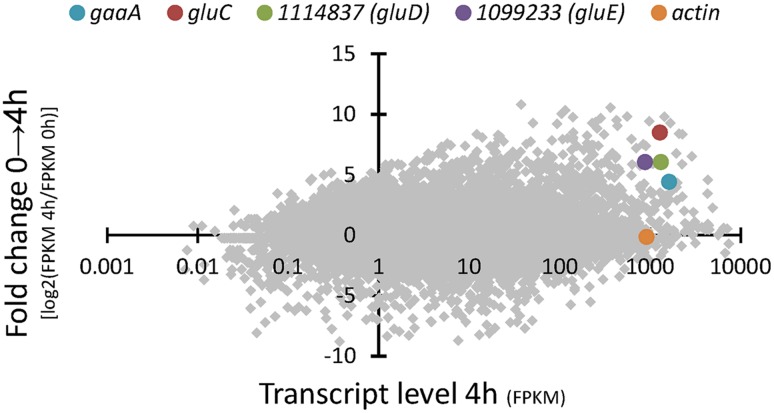
**RNA sequencing of *A. niger* wild type strain – fold change in transcript abundancies 4 h after the shift to D-glucuronic acid (*y*-axis) and transcript level (*x*-axis) 4 h after the shift to D-glucuronic acid**. The genes *gaaA*, *gluC*, actin and the genes with the protein IDs 1114837 (*gluD*) and 1099233 (*gluE*) are highlighted. Transcript levels are presented as fragments per kilobase of exon per million fragments mapped (FPKM). The protein ID numbers refer the numbers from the JGI MycoCosm *A. niger* ATCC 1015 v.4.0 database.

**FIGURE 3:**
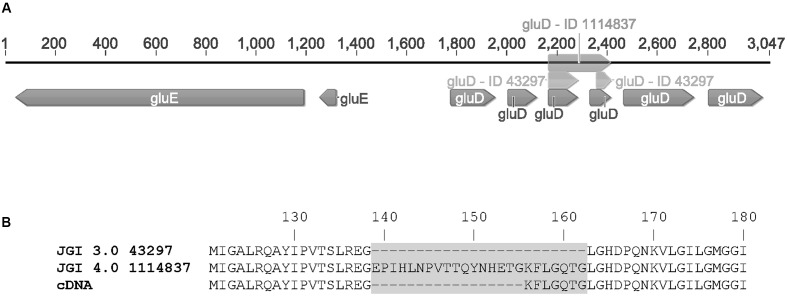
**(A)** The gene cluster of *gluD* (as predicted in JGI MycoCosm *A. niger* ATCC 1015 database v3.0 = ID 43297; v4.0 = ID 1114837 and sequenced from cDNA = *gluD*) and *gluE* (ID 1099233) and **(B)** the differences in protein sequences of GluD as predicted in the v3.0 (43297), v4.0 (1114837) and determined from cDNA.

RNA sequencing of the *A. niger* wild type strain ATCC 1015 cultivated in D-glcUA as sole carbon source revealed several putative genes with induced transcription (**Figure 2**). **Figure 2** presents the induction of transcript levels between 0 and 4 hours (*Y*-axis) and the absolute transcript levels at 4 h (*X*-axis). We selected genes that were induced on D-glcUA (**Figure 2**, values on *Y*-axis clearly above 1), had absolute transcript levels around similar or higher than that of actin at 4 h (**Figure 2**, *X*-axis) and are predicted to code for a metabolic enzyme, such as oxidoreductases. The D-galUA/D-glcUA reductase *gaaA* and the 2-keto-L-gulonate reductase *gluC* were among the most induced genes as reported earlier (Kuivanen et al., 2016). In addition, two genes, with the protein identifiers 1114837 and 1099233 (JGI, MycoCosm, *A. niger* ATCC 1015 v.4.0 database), putatively encoding a D-isomer specific 2-hydroxy acid dehydrogenase and an alcohol dehydrogenase, respectively, were induced. These genes are clustered in the genome in opposite directions relative to each other and share a common promoter region of 455 bp (**Figure 3A**). The fold change in transcript levels after the shift to D-glcUA was exactly the same for these genes while 1114837 had slightly higher transcript abundancy (**Figure 2**).

### The Clustered Genes gluD and gluE Code for 2-Keto-L-Gulonate Reductase and L-Idonate 5-Dehydrogenase

The open reading frames of the two genes were cloned in multicopy yeast expression vectors, expressed in yeast and the crude cell extracts were tested for activity with a small library of sugars and sugar acids. Both enzymes showed activity towards L-idonate. The 1114837 had activity with NADP^+^ as a cofactor whereas the 1099233 had activity when the cofactor was NAD^+^. In the case of 1114837 we noticed that the open reading frame that was custom synthetized according to the open reading frame as predicted in the DOE JGI *A. niger* ATCC 1015 v3.0 database (the protein ID in v3.0 is 43297), did not result in an active protein, however, when the gene was amplified from *A. niger* cDNA the resulting protein was active. In the current DOE JGI *A. niger* ATCC 1015 v4.0 database the exon prediction has been changed, however, both of the predictions (v3.0 and 4.0) are wrong. The sequence of the 1114837 amplified from cDNA differs from the predicted sequences: In the prediction v3.0, 21 nucleotides were predicted to be an intron and are missing in the open reading frame whereas in the prediction v4.0 the exons 3, 4 and the intron between them are combined. This is shown in the **Figure 3A** (v3.0 = 43297 and v4.0 = 1114837) and the differences in the resulting protein sequences are shown in **Figure 3B**. The correct gene sequence was deposited at GenBank with the accession number KX443112.

The enzyme 1114837 showed, besides the activity with NADP^+^ and L-idonate, also activity with 2-keto-L-gulonate and NADPH as cofactor. This suggests that the enzyme is a NADPH dependent 2-keto-L-gulonate reductase. We named the gene *gluD*. The gene 1099233 had activity with NAD^+^ and L-idonate but did not show activity with 2-keto-L-gulonate and NADH. It showed, however, activity with 5-keto-D-gluconate and NADH. We conclude that the enzyme is a NAD^+^ dependent L-idonate 5-dehydrogenase. We named the gene *gluE*.

For the more detailed characterization, histidine tagged GluD and GluE proteins were produced in yeast and the kinetic parameters of the purified proteins were investigated. Purified GluD showed NADPH/NADP^+^ dependent oxidoreductase activity toward 2-keto-L-gulonate and L-idonate with the k_cat_ values of 21.4 and 1.1 s^-1^, respectively. The *K*_m_ values for the substrates were 25.3 and 12.6 mM, respectively. Purified GluE protein had strictly NAD^+^/NADH dependent oxidoreductase activity towards L-idonate and 5-keto-D-gluconate with the *k*_cat_ values of 5.5 and 7.2 s^-1^, respectively. The *K*_m_ values for the substrates were 30.9 and 8.4 mM, respectively. Kinetic parameters of GluD and GluE are presented in **Table 2** and **Supplementary Figure 1**.

**Table 2:** Kinetic parameters of the purified GluD and GluE proteins.

Protein	Substrate	*V*_max_ (μmol min^-1^ mg^-1^)	*K*_m_ (mM)	*k*_cat_ (s^-1^)	*k*_cat_/*K*_m_ (M^-1^ s^-1^)
GluD	2-keto-L-gulonate	34.9 ± 0.5	25.3 ± 1.2	21.4 ± 0.3	8.46^∗^10^2^
	L-idonate	1.7 ± 0.1	12.6 ± 0.8	1.1 ± 0.0	8.73^∗^10
GluE	L-idonate	7.6 ± 0.1	30.9 ± 1.5	5.5 ± 0.5	1.78^∗^10^2^
	5-keto-D-gluconate	10.0 ± 0.0	8.4 ± 0.1	7.2 ± 0.0	8.57^∗^10^2^

*Data represent means ± standard deviation from three technical repeats*.

### Deletion of gluD or gluE has an Effect on D-Glucuronic Acid Catabolism

We also deleted the genes *gluD* and *gluE* from *A. niger* and tested the resulting phenotypes. For the *gluD* gene deletion CRISPR technology was used to remove the native gene. This was implemented using the *AMA*-plasmid expressing Cas9 (Nodvig et al., 2015), an *in vitro* synthetized sgRNA and the deletion cassette with the selectable marker *pyrG*. *GluE* gene was deleted without CRISPR using only the deletion cassette containing *pyrG* marker. Both of the gene deletions were confirmed with diagnostic PCR and the mutant strains were tested for growth and ability to catabolize D-glcUA.

**FIGURE 4:**
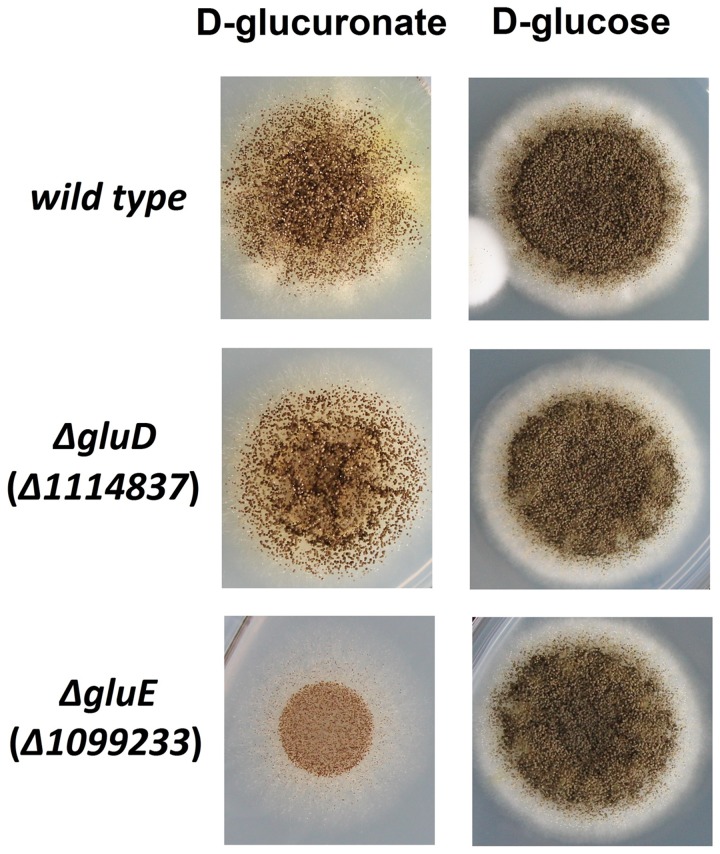
**Growth of the *A. niger* strains *wt*, Δ*gluD*, and Δ*gluE* on agar plates with D-glucuronic acid or D-glucose as sole carbon source**.

**FIGURE 5:**
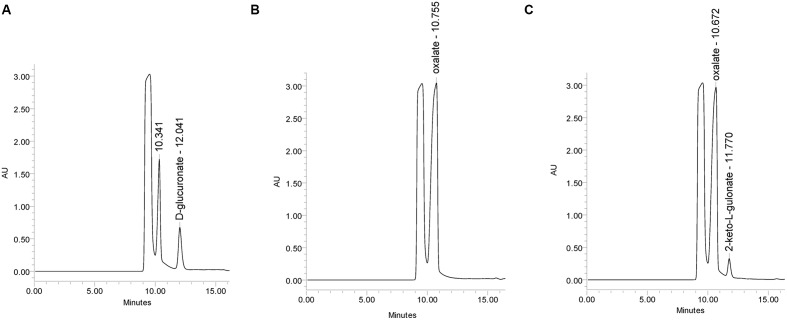
**HPLC analysis of the *A. niger* growth medium (A)** at 0 h, **(B)** at 72 h with the *A. niger* wild type strain, and **(C)** with Δ*gluD*.

The mutant strain Δ*gluD* did not show reduced growth when cultivated on agar plate with D-glcUA as sole carbon source (**Figure 4**). However, in the liquid cultivation on D-glcUA, a phenotype was observed for Δ*gluD*: 2-keto-L-gulonate accumulated in the medium after D-glcUA was consumed (**Figures 5A,C**). This was not observed with the wild type strain (**Figure 5B**). In the case of the mutant strain Δ*gluE*, growth on D-glcUA plate was reduced (**Figure 4**). In addition, the consumption of D-glcUA in liquid cultivation was almost completely disrupted in Δ*gluE* (**Table 3**).

**Table 3:** Concentration of D-glucuronic acid (D-glcUA) in submerged cultivations by the *A. niger* wild type strain (*wt*) and Δ*gluE*.

	D-glcUA (g l^-1^)		
Strain	0 h	20 h	50 h
*wt*	20.4 ± 00	12.0 ± 0.4	0.0 ± 0.0
Δ*gluE*	20.4 ± 00	19.1 ± 0.1	17.8 ± 0.2

*Data represent means ± standard deviation from three biological repeats*.

## Discussion

D-GlcUA is a biomass component that is catabolised by many microorganisms including fungi. However, the catabolic pathway in fungi is only partly known. Recently, we identified the gene *gluC* that is essential for D-glcUA catabolism in the filamentous fungus *A. niger* (Kuivanen et al., 2016). The gene encoded an enzyme reducing 2-keto-L-gulonate to L-idonate using NAD^+^ as cofactor. We also showed that the *gaaA* gene encoding a D-galUA and D-glcUA reductase is induced on both substrates, D-galUA and D-glcUA. All this indicates that D-glcUA is first reduced to L-gulonate, then converted to 2-keto-L-gulonate by an unknown mechanism, and then reduced to L-idonate by the GluC. In the present study, we identified a gene cluster that is involved in D-glcUA catabolism in *A. niger* consisting of the genes *gluD* and *gluE*. In this cluster, *gluD* encodes a NADP^+^ dependent enzyme that, similar to GluC, catalyzes the reaction between 2-keto-L-gulonate and L-idonate. The other gene in the cluster, *gluE*, encodes a NADH dependent enzyme that catalyzes the reaction between L-idonate and 5-keto-D-gluconate. The latter reaction catalyzed by GluE seems to be the next step after the formation of L-idonate in the catabolic D-glcUA pathway in *A. niger*. This, still uncomplete pathway is summarized in the **Figure 1C**.

The D-glcUA pathway genes *gluD* and *gluE* are clustered in a similar manner as the D-galUA catabolic pathway genes *gaaA* and *gaaC* (Martens-Uzunova and Schaap, 2008) in the *A. niger* genome. An ortholog of *gluD-gluE* gene cluster is present in most of the sequenced aspergilli (AspGD)^2^. In fungi, genes of the same metabolic pathway are sometimes co-localized on chromosomes, i.e., they form chromosomal clusters (Wisecaver et al., 2014). What drives the formation of these clusters is debated. The need to ensure removal of toxic intermediates (McGary et al., 2013) has been proposed as the ultimate reason, but mere transcriptional co-regulation (Gordon et al., 2015) might have other benefits too. In this case, *gluD* and *gluE* share a common promoter and transcription of the genes is induced with a similar pattern on D-glcUA. Thus, transcriptional co-regulation is a possible explanation for the formation of *gluD-gluE* cluster. It is also suggested that soil-dwelling fungi may have obtained genes from bacteria for catabolism of unusual carbon sources through horizontal gene transfer (Wisecaver et al., 2014; Wisecaver and Rokas, 2015). In fact, it was suggested that fungal β-glucuronidase genes are derived from bacteria allowing fungi to hydrolyse glucuronides resulting in access to released monomeric D-glcUA (Wenzl et al., 2005). In bacteria, metabolic genes are often present in clusters such as in the case of catabolic L-idonate pathway in *E. coli* (Bausch et al., 2004). If such metabolic genes are acquired from bacteria via horizontal gene transfer, it may eventually lead to the formation of metabolic gene clusters in fungi as well.

In the previous study, deletion of *gluC* in *A. niger* disrupted the D-glcUA catabolism nearly completely (Kuivanen et al., 2016). Even though, GluC and GluD catalyze the same reaction and both genes are induced on D-glcUA, it seems that GluD cannot compensate the loss of GluC activity in the fungal D-glcUA pathway (deletion of *gluC* disrupted growth on D-glcUA; Kuivanen et al., 2016). This might be due to cofactor requirements: GluC requires NADH and GluD NADPH. In fact, it is surprising and unusual that two enzymes, in this case GluC and GluD, are present for the same reaction, but have different cofactor requirements. Since both reactions are reversible a possible interpretation is that the enzyme couple may act as an NAD(P)^+^ transhydrogenase adjusting the ratio of NAD^+^/NADH and NADP^+^/NADPH. Deletion of *gluD* did not result in reduced or no growth on D-glcUA as sole carbon source. However, it resulted in a phenotype of accumulating 2-keto-L-gulonate when cultivating on D-glcUA. This observation further supports the hypothesis that the fungal catabolic D-glcUA pathway proceeds through the oxidation of L-gulonate to 2-keto-L-gulonate. The oxidation of L-gulonate to 2-keto-L-gulonate is a biochemical reaction that is not described in the literature and the responsible enzyme in *A. niger* still remains unclear. In the case of 2-keto-L-gulonate reductase activity, an unspecific bacterial D-gluconate 2-dehydrogenase (EC 1.1.1.215) had been described that showed also activity for the reaction between L-idonate and 2-keto-L-gulonate (Yum et al., 1998). This bacterial enzyme used NADP^+^/NADPH as a cofactor similar to the GluD described in this study. However, we conclude that GluD is the first specific NADPH dependent 2-keto-L-gulonate reductase reported to date.

The protein product of the gene *gluE*, described in this study, catalyzed the reversible reaction from L-idonate to 5-keto-D-gluconate using NAD^+^/NADH as cofactor. A similar enzyme activity has been described in the filamentous fungus *Fusarium* sp. already in Takagi (1962), however, this enzyme activity was strictly NADP^+^/NADPH dependent. In plants, an NAD^+^/NADH enzyme (EC 1.1.1.366) oxidizing L-idonate to 5-keto-D-gluconate functions in the pathway converting L-ascorbic acid to L-tartaric acid (DeBolt et al., 2006). In addition, *E. coli* has an L-idonate 5-dehydrogenase, IdnD (EC 1.1.1.264), producing 5-keto-D-gluconate from L-idonate with NAD^+^ as cofactor in the catabolic L-idonate pathway (Bausch et al., 1998). GluE has only low sequence homology toward the other characterized L-idonate 5-dehydrogenases and it is the first reported fungal NAD^+^ dependent L-idonate 5-dehydrogenase. The *gluE* deletion in *A. niger* had also a phenotype – growth was reduced and D-glcUA consumption was ceased. This is a strong indication that the gene is part of the fungal catabolic D-glcUA pathway and the pathway passes through the oxidation of L-idonate to 5-keto-D-gluconate.

It is unclear how the fungal D-glcUA pathway continues after formation of 5-keto-D-gluconate. In plants, a NAD^+^ dependent L-idonate 5-dehydrogenase forming 5-keto-D-gluconate was described (Wen et al., 2010). This was suggested to be part of the pathway for L-ascorbic acid degradation (DeBolt et al., 2006). In this pathway, the resulting 5-keto-D-gluconate is split by an aldolase to L-threo-tetruronate and glycolaldehyde. The L-threo-tetruronate is then oxidized to L-tartaric acid. In this pathway, only the L-idonate 5-dehydrogenase gene had been identified. Another possibility would be a route similar to the L-idonate catabolism in bacteria. In *E. coli*, a NAD^+^ specific L-idonate 5-dehydrogenase is reducing the L-idonate to 5-keto-D-gluconate and the 5-keto-D-gluconate is subsequently reduced to D-gluconate (Bausch et al., 2004). D-Gluconate is then phosphorylated and the resulting 6-phosphogluconate enters the Entner–Doudoroff pathway. If 5-keto-D-gluconate is reduced to D-gluconate in the fungal D-glcUA pathway in *A. niger*, it would connect D-glcUA catabolism with the catabolism of D-glucose. *A. niger* oxidizes extracellular D-glucose to D-gluconate which is then taken up and catabolized further through the phosphorylation to D-gluconate-6-phosphate and subsequently via pentose phosphate pathway (Muller, 1985). It is also suggested that some strains of *A. niger* catabolize D-gluconate through the non-phosphorylative Entner–Doudoroff pathway including dehydratation of D-gluconate to 2-keto-3-deoxy-gluconate which is the split to D-glyceraldehyde and pyruvate by the action of an aldolase (Elzainy et al., 1973; Allam et al., 1975). However, the fate of 5-keto-D-gluconate in the fungal D-glcUA pathway still remains to be unraveled.

## Author Contributions

JK and PR designed and JK carried out all the experimental work and analyzed the data. MA processed and analyzed the RNAseq data. JK and PR drafted the manuscript. PR designed the fundamental concept and participated in the coordination of the study. All the authors read and approved the final manuscript.

## Conflict of Interest Statement

The authors declare that the research was conducted in the absence of any commercial or financial relationships that could be construed as a potential conflict of interest.

## Footnotes

1 www.ic50.tk

2 http://aspgd.broadinstitute.org/cgi-bin/asp2_v3/shared/show_protein_cluster.cgi?site=asp2_v9\&id=2050676

## Supplementary Material

FIGURE S1 **Oxidoreductase activity of purified GluD towards (A)** 2-keto-L-gulonate and **(B)** L-idonate with NADPH and NADP^+^, respectively and oxidoreductase activity of purified GluE toward **(C)** L-idonate and **(D)** 5-keto-D-gluconate with NAD^+^ and NADH, respectively. Data represent means ± standard deviation from three biological repeats. If error bars not visible are smaller than the symbol.
