# Supplementary figures and images for: Clustered Genes Encoding 2-Keto-l-Gulonate Reductase and l-Idonate 5-Dehydrogenase in the Novel Fungal d-Glucuronic Acid Pathway
Source: Front Microbiol. 2017 Feb 14;8:225. doi: 10.3389/fmicb.2017.00225 (PMC5306355; doi:10.3389/fmicb.2017.00225)

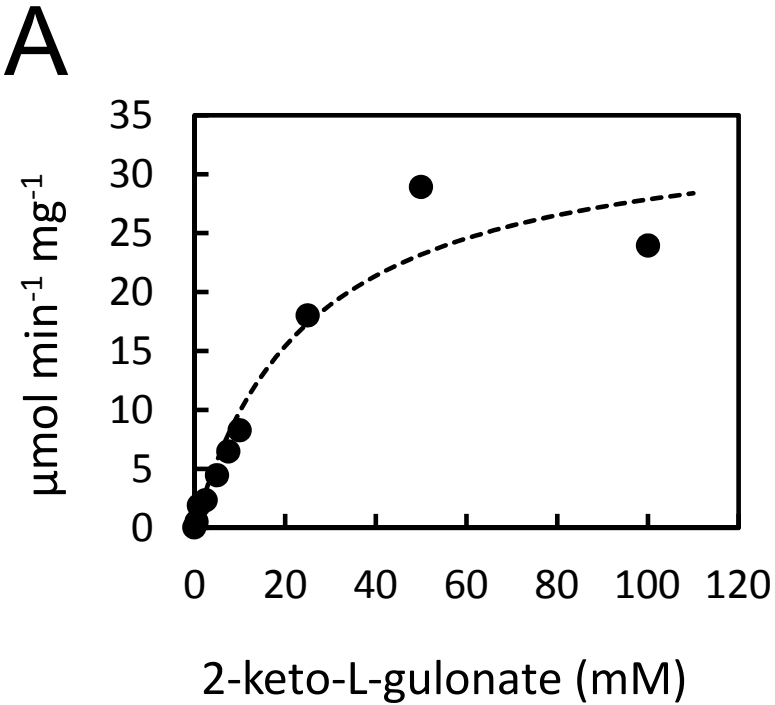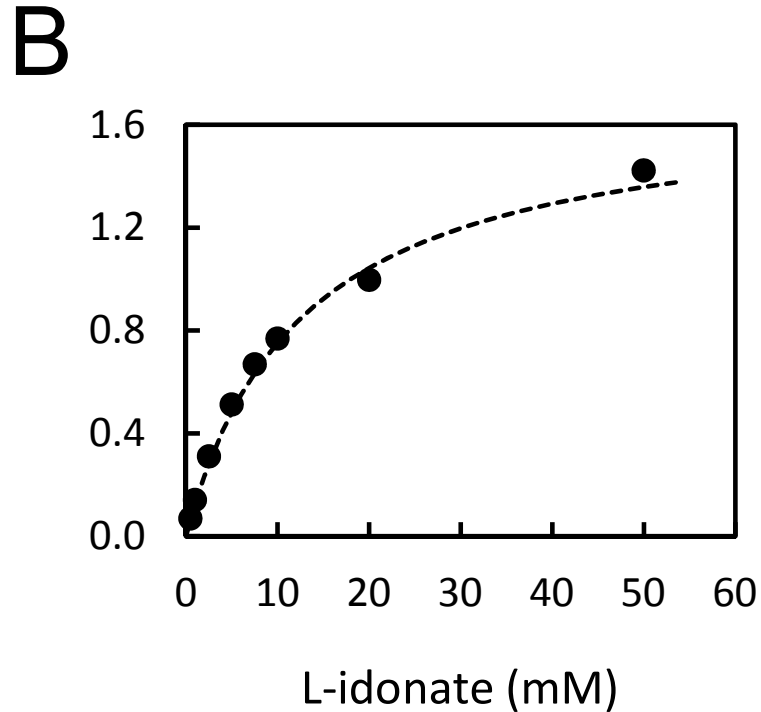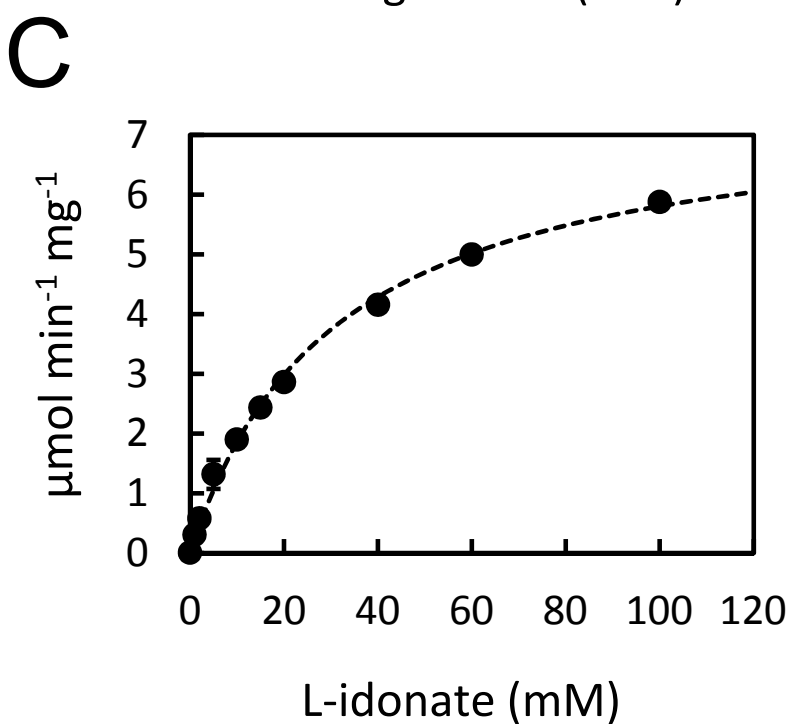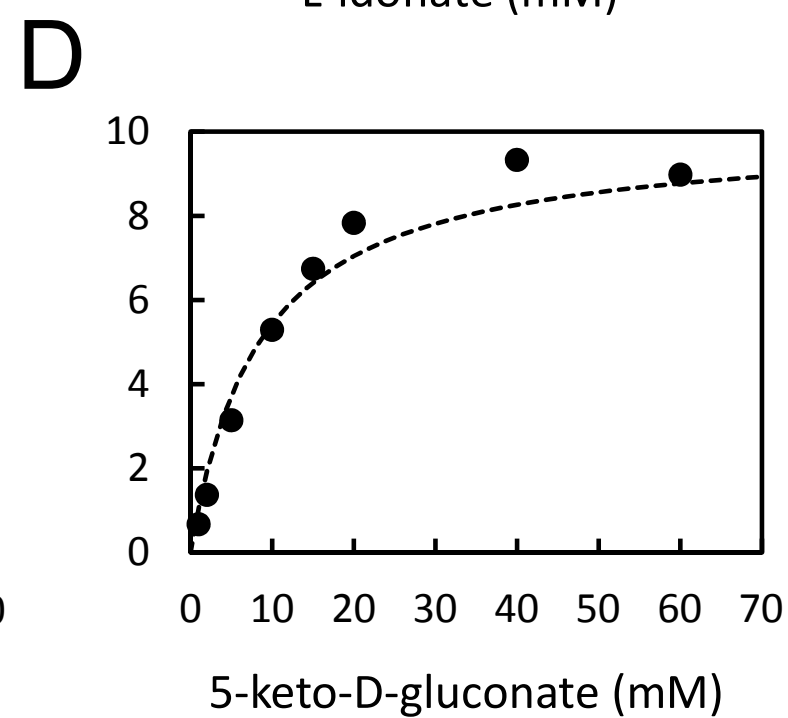

Supplement: FIGURE S1 — Oxidoreductase activity of purified GluD towards (A) 2-keto-L-gulonate and (B) L-idonate with NADPH and NADP+, respectively and oxidoreductase activity of purified GluE toward (C) L-idonate and (D) 5-keto-D-gluconate with NAD+ and NADH, respectively. Data represent means ± standard deviation from three biological repeats. If error bars not visible are smaller than the symbol. [file Image_1.PDF]
